# Mushroom: an emerging source for next generation meat analogues

**DOI:** 10.3389/fnut.2025.1638121

**Published:** 2025-09-11

**Authors:** Jibanjyoti Panda, Pinku Chandra Nath, Awdhesh Kumar Mishra, Sarvesh Rustagi, Debasis Nayak, Renald Blundell, Yugal Kishore Mohanta

**Affiliations:** ^1^Bioresource and Traditional Knowledge Laboratory, Department of Wildlife and Biodiversity Conservation, Maharaja Sriram Chandra Bhanja Deo University, Mayurbhanj, Odisha, India; ^2^Research and Development Cell, Manav Rachna International Institute of Research and Studies (Deemed-to-be-University), Faridabad, Haryana, India; ^3^Department of Biotechnology, Yeungnam University, Gyeongsan, Gyeongsangbuk-do, Republic of Korea; ^4^Department of Food Technology, School of Agriculture, Maya Devi University, Dehradun, Uttarakhand, India; ^5^Department of Physiology and Biochemistry, University of Malta, Msida, Malta; ^6^Nano-Biotechnology and Translational Knowledge Laboratory_,_ Department of Applied Biology, University of Science and Technology Meghalaya, Ri-Bhoi, Meghalaya, India; ^7^Centre for Herbal Pharmacology and Environmental Sustainability, Chettinad Hospital and Research Institute, Chettinad Academy of Research and Education, Kelambakkam, Tamil Nadu, India

**Keywords:** edible mushroom, meat analogues, sustainable food products, quality aspects, consumer perception

## Abstract

**Background:**

In recent years, plant-based and alternative protein sources have garnered attention. Since they may resemble the texture, flavour, and nutritional profile of typical meat products, mushroom-based meat substitutes have received attention. However, scaling up production, cost-effectiveness, and nutritional requirements similar to animal-based meat products remain hurdles. Thus, understanding these dynamics is crucial to the global development and adoption of next-generation mushroom-based meat substitutes.

**Scope and approach:**

This review examined and synthesised the current mushroom-based meat analogue research, concentrating on their physicochemical, nutritional, and qualitative properties. Also, evaluated worldwide market viability, consumer acceptance, and development and adoption difficulties and potential for next-generation mushroom-based meat substitutes.

**Key findings and conclusions:**

Due to their fleshiness, mushrooms can replace beef in sausages, nuggets, and patties. Rising vegetarianism and health concerns require meat substitutes. Due to their easy cultivation, excellent nutritional value, low fat and calorie content, and steady growth, mushrooms can meet this demand. However, there are still numerous chances and challenges to improve sensory features (texture, taste, and flavour), optimise processing, assess consumer satisfaction, and use different medicinal mushrooms as meat replacements. Thus, they are essential to the creation of nutritious, sustainable meat-based foods.

## Graphical abstract

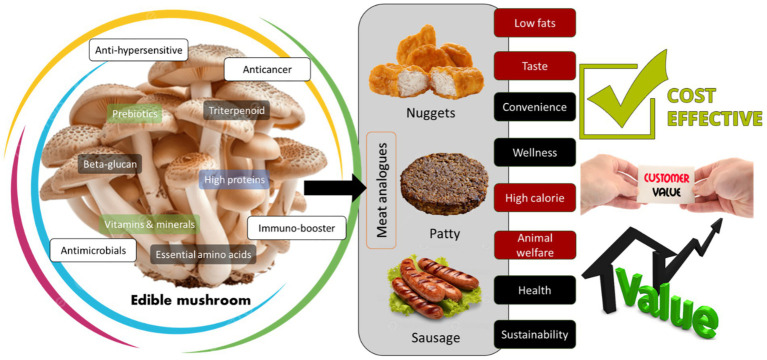

## Highlights

- Edible mushroom-based meat analogues exceed plant and animal-based options.
- Promoting sustainable edible mushroom quality improves product acceptance.
- Consumers have a positive impression of mushroom-based meat alternatives.
- Edible mushroom-based meat analogue sectors are growing worldwide.

## 1 Introduction

The ongoing rise in population growth results in the challenge of satisfying the requirements for high-quality food and attaining nutritional security. Experts predict that the rapid growth of the world’s population will surpass 9 billion people within the next 25 years (1). The process of urbanisation and the rapid increase in population lead to a significant need for the consumption of meat and processed food that is rich in nutrients (2, 3). Foods, such as meat and fish, are crucial components of the daily diet for many individuals because of their appealing sensory characteristics and nutritional qualities, which encompass abundant amounts of high-quality proteins, vitamins, and minerals. Furthermore, consuming processed foods has been connected to a number of long-term health issues, including an increase in obesity, diabetes, heart disease, malignancies, hypertension, and many more. Furthermore, it poses a threat to aquatic environment and contributes to other environmental issues like greenhouse gas emissions. In addition, consuming animal meat may expose one to veterinary antibiotics and increase the risk of zoonotic diseases (4, 5).

Now-a -days, consumers are changing their eating habits to include healthier options as they learn more about the possible connections between nutrition and health. Incorporating more nutrients like vitamins, minerals, and nutraceuticals into one’s diet and decreasing consumption of chemicals like sugar, salt, and saturated fat are the principles of a healthy diet. Sticking to these eating habits helps one keep a healthy weight and lowers the risk of certain lifestyle-related diseases (6, 7). Therefore, there is a growing need for easy-to-digest, nutritious foods. As a result, there is a movement to reduce the amount of meat and other animal products eaten by humans. A flexitarian, vegetarian, or vegan diet that restricts meat consumption can help with this. Anyone looking to eat meat while also making a sustainable and eco-friendly dietary shift can benefit from this method (8, 9).

**Figure 1:**
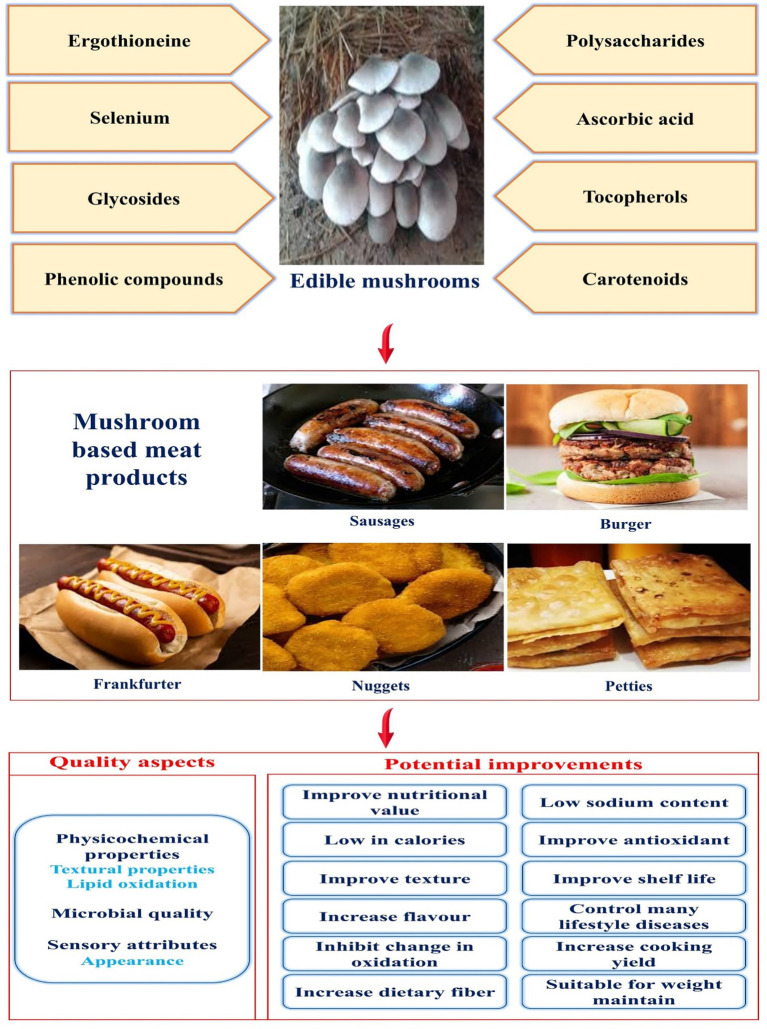
Role of edible mushrooms as meat analogues and their impacts.

Therefore, all of these components focus on developing substitutes for animal-derived meat through non-animal food production techniques, such as meat or mead-based products derived from mushrooms. Some of the nutraceutical found in edible mushrooms are β-glucan, dietary fibres, bioactive peptides, terpenes, glycoproteins, alcohols, minerals, phenolic compounds, tocopherols, unsaturated fatty acids, vitamin D, and ascorbic acids (Figure 1). These substances show hypoglycemia, antioxidant, antimicrobial, anti-inflammatory, anti-viral, antihypertensive, hypolipidemic, and anticancer properties (10–12). Because of their chewy texture and meaty flavour, mushroom-based processed foods and meat analogues are increasingly recognised as foods and supplements. Additionally, in line with the UN’s Sustainable Development Goals (SDGs), is the concept of substituting mushrooms for meat. A mycoprotein, derived from mushrooms, is emerging as a sustainable and healthy substitute, providing high-quality protein, necessary amino acids, and fibre, while being low in fat and calories, and enhancing digestion (13, 14). The elements listed above concentrate on developing substitutes for animal meat by using nonanimal sources, such as mushrooms. Meat analogues made of mushrooms addresses social, cultural, and animal welfare concerns while providing health advantages not present in regular meat. These issues are forcing the food industry to create and develop more sustainable and healthful products (15, 16). Comparing edible mushroom-based analogues with fermentation-based mycoproteins is shown in Table 1. The research on mushroom-derived meat substitutes introduces an innovative method for sustainable and functional food creation by employing edible fungi as the main component. Mushrooms have a natural umami flavour, a fibrous texture, and a wealth of bioactive ingredients, such as antioxidants and B-vitamins, distinguishing them from traditional plant-based meats. This review discusses the latest trends in mushroom-based meat analogues by focusing on their physicochemical characteristics, nutritional composition, and overall quality attributes. Additionally, explored the potential of mushroom-based meat analogues in the global market, considers factors influencing consumer acceptance, and analyses both the limitations and opportunities associated with developing and adopting next-generation mushroom-based meat analogues. This review also highlights clean-label formulations, and nutritional enhancement devoid of synthetic ingredients, employing various methodologies. Moreover, it conforms to circular economy principles by facilitating both environmental and social effect. The study distinctly enhances the alternative protein sector by meeting environmental, health, and consumer needs, establishing mushrooms as a promising area in meat substitute innovation.

**Table 1:** Comparison of mushroom-based substitutes and fermentation-derived mycoproteins.

Characteristics	Mushroom-derived alternatives	Mycoproteins
Biological origins	Fruiting bodies of edible mushrooms (e.g., *Pleurotus ostreatus*, *Agaricus bisporus*)	Filamentous fungi (*Fusarium venenatum*) grown via fermentation
Portion of organism utilised	The mushroom itself (harvested and processed)	The mycelium (root-like structure) cultivated in bioreactors
Production techniques	Slicing, seasoning, harvesting, and perhaps some light processing	Fermentation on a large scale under regulated circumstances
Texture and structure	Natural fibrous texture, similar to meat when cooked	Moulded, fibrous structure created by fungal hyphae (somewhat meat-like)
Processing level	Generally low-processed	Highly processed, often mixed with binders and flavours
Nutritional profile	Moderate protein, fibre, antioxidants, B vitamins	High-quality protein, fibre (mostly β-glucans), low fat
Regulatory status	Treated as traditional food in many regions	Often classified as novel food, needs safety assessments

## 2 Nutritional profile of mushroom meat alternatives with animal-based meat analogue

Based on information about the nutrients found in meat, egg, mushrooms have special nutritional qualities (Table 2). Utilising mushrooms as meat analogues provides benefits such as a naturally meaty texture and umami flavour, in addition to being a sustainable and nutrient-dense alternative to conventional meat substitutes. Mushrooms have a higher protein content than wheat and are comparable to, or even greater than, meats from cattle and fowl, such as pig and beef. Mushrooms have a perfectly balanced amino acid content, and also contain a wide variety of amino acids (17–19). Mushrooms are unequivocally devoid of cholesterol and low in fat, with a substantial proportion of their fat content comprising unsaturated fatty acids, which are advantageous for health (20). While animal fats have more saturated fatty acids, they also include some unsaturated fatty acids. Increased blood lipid levels brought on by overconsumption of animal fats may cause atherosclerosis and coronary heart disease to develop (21). Substituting mushrooms for meat can satisfy the body’s requirement for unsaturated fatty acids and reduce the adverse effects of consuming excessive amounts of saturated fatty acids. A good source of vitamin B_12_ is animal-based foods, including meat, eggs, and milk. Vitamin B_3_ and other B vitamins are abundant in mushrooms; in fact, their concentrations often exceed those of beans and meat (22). Proteins from soybeans and wheat cause common allergies. Although soy can trigger allergic reactions in some individuals, pea protein has shown no such adverse effects. As a result, plant-based meat alternatives are increasing the use of pea protein, which is displacing soy protein (23). On the other hand, allergies effects of mushrooms are quite rare and also it simulate the flavour of flesh in part because of their high fibre content and fibrous structure (5). Researcher were able to improve the quality of a plant-based meat alternative by using soy protein as the primary component and incorporating a small quantity of flat mushrooms (24). This process produced a meat product with a more appealing fibrous structure. Current biomedical research indicates that the organism’s overall protein utilisation will drop dramatically if it does not have a required amino acid in the acquired amino acid fraction. The mushrooms discovered in China display an extensive range of species, each possessing different protein concentrations and amino acid compositions. Nevertheless, it is crucial to acknowledge that the proportion of amino acids in a particular species is generally inadequate to fulfil the dietary needs of the human body. Using the differences in the mass fraction of essential amino acids and the amino acid ratio coefficient between different species of mushrooms or other foods might enhance the body’s bio-utilisation of protein. This will significantly improve the usage rate, quality, and nutritional content of mushroom protein.

**Table 2:** Nutritional composition of meat, mushrooms, and other popular plant-based meat substitutes^1^.

Source type	Species/source	Carbohydrates (g/100 g)	Protein (g/100 g)	Fat (g/100 g)	Cholesterol (mg/100 g)	Energy (kJ/100 g)
Mushroom	*Agrocybe aegerita,* dried	56.1	23.1	2.3	0	1,304
	*Auricularia auricula,* dried	65.6	12.1	1.5	0	1,376
	*A. auricula,* water-swollen	6	1.5	0.2	0	135
	*Agaricus bisporus,* fresh	31.6	38.7	3.3	0	1,317
	*Flammulina velutipes,* fresh	6	2.4	0.4	0	158
	*Lentinula edodes,* dried	61.7	20	1.2	0	1,433
	*Pleurotus eryngii,* fresh	8.3	1.3	0.1	0	167
	*P. ostreatus,* dried	4.6	1.9	0.3	0	122
	*Volvariella volvacea,* dried	4.3	2.7	0.2	0	126
Egg	Egg	2.8	13.3	8.8	585	599
Meat	Beef	2	19.9	4.2	84	528
	Chicken	9.4	19.3	1.3	106	698
	Grass carp	Nil	16.6	5.2	86	475
	Large yellow croaker	0.8	17.7	2.5	86	404
	Pork	2.4	13.2	37	80	1,634
	Shrimp (kiwai shrimp)	3.9	18.2	1.4	181	427
Plant based	Pea, dried	68.8	20.3	16	0	1,504
	Soyabean, dried	34.2	35	16	0	1,768
	Lentils, boiled	20	9	0.4	0	485.34

1 Source: Date obtained from Food Composition Database, Food Nutritional Composition Search, https://nlc.chinanutri.cn/fq/.

## 3 Potential techniques involved with mushroom-based meat analogues

**Figure 2:**
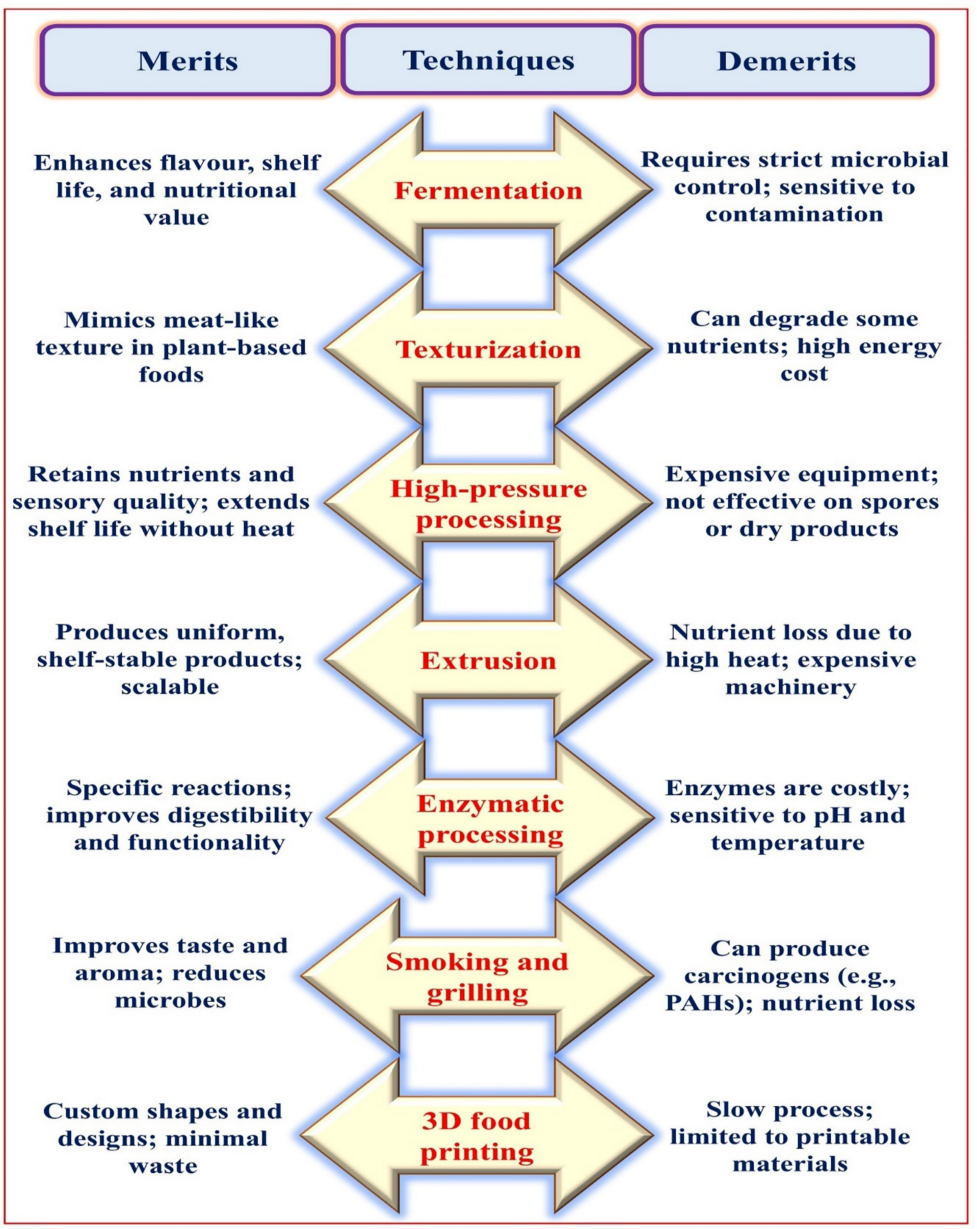
Some of the merits and demerits associated with the techniques involved in mushrooms-based meat analogues.

Mushrooms are a great meat substitute because of their meaty, fibrous texture. The fibrous component chitin, which is present in the cell walls of mushrooms and contributes to their hardness and chewiness, is responsible for this texture (25, 26). Mushrooms maintain their shape when cooking because to their fibrous nature, which ensures a robust texture and satisfying chew. For mushroom-based products like nuggets and burgers, this is crucial since the texture gives the impression of meat, making them more appealing to flexitarians and vegetarians (15, 27). Products made with mushrooms have their texture and taste improved by technological advancements in the food industry, which include texturization, extrusion, fermentation, high-pressure processing (HPP), enzymatic processing, smoking, and grilling. In addition, techniques like vacuum frying and freeze-drying can create unique flavours and sensations without compromising nutritional value. Light and crunchy delicacies with retained flavour are the result of freeze-drying mushrooms, which effectively removes moisture while preserving their structure and nutrition. Vacuum drying, on the other hand, allows for lower-temperature frying, which reduces oil absorption and nutrient degradation (15). In addition to this, these potential techniques have some merit as well as demerit which plays vital role for their sustainable uses (Figure 2).

- **Fermentation:** Utilises microorganisms to decompose sugar into advantageous chemicals such as acids, gases, or alcohol.
- **Texturization:** To create a texture similar to that of fibrous meat, scientists alter the physical properties of mushrooms. Agar, xanthan gum, and carrageenan are hydrocolloids that help mushrooms hold water and form gels, which makes the mushroom structure denser and more cohesive. Products made from mushrooms benefit from heat treatment techniques like steaming, blanching, or baking because it breaks down cell walls, releases natural sugars, and enhances texture and flavour.
- **Extrusion:** The hydrothermal method uses a die to apply high pressure and heat, turning mushroom combinations into fibrous, meat-like textures. Amino acids and reducing sugars undergo the Maillard reaction when heated during extrusion, which improves flavour development and creates complex scents like as meat products.
- **HPP:** An innovative non-thermal preservation method that enhances food safety and extends the shelf life of the products by inactivating enzymes and microorganisms under pressure, while maintaining nutrients and sensory attributes to a minimal extent. HPP-treated mushroom powder exhibits reduced viscosity, increased fluidity, and greater solubility of proteins and polysaccharides, rendering it suitable for convenience foods and additives.
- **Enzymatic processing**: Modify the structural components of mushrooms using particular enzymes to enhance their texture, flavour, and nutritional profile. Lipases and proteases, for example, degrade proteins and lipids to produce amino acids and fatty acids.
- **Smoking and grilling:** Conventional techniques bestow mushrooms with a smoky, burnt flavour, augmenting their umami characteristics and strengthening their attractiveness as meat alternatives. Enhance mushroom texture to get a harder, meat-like consistency, providing a gratifying bite reminiscent to grilled foods.
- **3D food printing:** A technique for additive manufacturing that allows for the customisation of esthetical and nutritional aspects, therefore decreasing production time and material waste.

## 4 Mushroom based meat analogues

The term “meat analogues” refers to plant-based alternatives to meat that are highly convincing in appearance, taste, and feel. Because of their longer shelf-life, cheaper production costs, and decreased vulnerability to seasonal supply changes, these particular food items provide economic advantages to food firms. In comparison to burgers, analogues had fewer calories, less saturated fat and sodium, no cholesterol, and far more fibre by weight (18, 28). As a result, people view them as a trendy and health-conscious meal option. Proteins (20–50%), lipids (1–5%), and polysaccharides (2–30%) are some of the many beef components that give the meat its distinctive texture and flavour (29). The three main types of meat alternatives are those derived from plants, those grown in cells, and those created by myco-organisms, such as edible mushrooms and other types of fungus.

Mushrooms are regarded as a valuable nutritional item derived from fungi, known for their abundant supply of beneficial compounds. Mushrooms are a practical choice as a meat alternative due to their high protein content, beneficial bioactive compounds, low fat, and sodium levels (7, 30). Furthermore, the substantial amounts of glutamic acid, aspartic acid, and ribonucleotides in mushrooms contribute to their distinct umami flavour, associated with a brothy, savoury, rich, or meaty taste impression (31). Moreover, a process forms cross-linked structures in the extruded meat substitutes, resulting in enhanced antioxidant properties. This is because mushrooms contain polyphenols and protein interactions. The significant increase in mushroom production in recent years, feasible to manufacture large quantities of meat substitutes using mushrooms. In comparison to other non-meat protein sources, there are additional reasons to prefer mushroom protein. In addition, mushrooms contain a diverse range of nutraceuticals that provide numerous health benefits to consumers. Researchers have also discovered that mushrooms possess antibacterial properties, which can significantly extend the shelf-life of processed meat substitutes. The mushroom species often used as alternatives to beef, crab, and chicken meat include white button mushrooms, agaricus, shiitake, portobello, chanterelle, and enoki mushrooms (32). Mushrooms include dietary fibres that contribute to several physical properties, including texture, stability, emulsification, thickening, and gelling. Substituting mushrooms for textured soy protein resulted in a notable improvement in the sensory characteristics of the meat substitute (nuggets) (8). Research has enhanced the texture and antioxidant properties, particularly the ability to scavenge DPPH and the phenolic activity, of a meat substitute in their research. To accomplish this, they integrated oyster mushrooms into a blend of full-fat soy, wheat gluten, and corn starch in varying proportions (4, 8, and 12%). They employed the ingredients in a ratio of 0.5, 0.4, and 0.1 (33). It was found that increasing the proportion of mushrooms significantly improved the specific characteristics of the meat substitute. Development of a plant-based meat substitute by blending oyster mushrooms with soy protein has been reported (34). Researchers have formulated various meat analogues using mushrooms, such as salted cooked beef, chicken sausages, tuna, kuruma shrimp, classic Turkish meatballs, fermented pork sausages, sutchi catfish patties, and emulsion-type pork sausages (1) (Table 3).

**Table 3:** Edible mushrooms and their contributions to functional meat-based food products.

Mushrooms	Food products	Ingredients used	Impact on foods	References
*A. bisporus*	Beef meat emulsion	2% of MP	Changes in texture were noticeable, enhanced viscoelasticity, emulsion strength and tolerance to high temperatures. Increased protein adsorption at the lipid interface and leading to a more stable emulsion.	(107)
*A. bisporus*	Sutchi catfish patties (*Pangasius hypophthalmus*)	15% MP	Improvements in dietary value have been made, reducing the rate at which lipid breakdown progressed. The total volatile nitrogen in the base was drastically reduced. Fewer total plates in the blood. Longer storage time (up to 16 days) than the control group.	(45)
*A. bisporus*	Beef burger	0.5–30% MP	Changed the consistency, dampness, and water-activity levels. Hardness was much lower than the norm. Not a problem with the product’s coloration.	(82)
*A. bisporus*	Beef patties	1, 2% or 4%	Reduced production of oxidised lipid molecules. Compared to the controls, malondialdehyde, and volatile aldehydes were reduced. Increased protein oxidation products at increasing concentrations, while decreasing thiol and tryptophan fluorescence loss at 1% inclusion. Concentration-dependent storage time extension.	(108)
*A. bisporus*	Ground beef (80/20 blend)	10, 20, 30, 40, or 50%	There was no change in output, satisfaction ratings, brightness, or perceived redness. Boosted the saturation and yellowness. Lowered the end items’ mechanical characteristics, salt content, and fat content. The potential exists to lower salt levels, making meat products a healthier option.	(109)
*A. bisporus*	Steamed meat items	0–40% MP	Enhance the qualities of the senses, the binding, and the texture.	(62)
*A. bisporus*	Meat cake	Mycelia 27%	Improve the material’s texture and the general acceptance.	(84)
*A. bisporus*	Sausage	83.5% MP	Increase yield, emulsion ability, and textural quality.	(32)
*Boletus edulis*	Beef burger/ frankfurters	1, 3, or 5% extract	Defended against the oxidation of fats. Eicosapentaenoic (c20:5n3) and arachidonic (c20:4n6) acids are encapsulated for safety. Prolonged storage time.	(110)
*Calocybe indica*	Meat nuggets	0–27% MP	Improve the sensory qualities.	(111)
*Coprinus comatus*	Fermented sausages	15% MP	There was an improvement in the aroma profile, taste, flavour, and texture.	(112)
*F. velutipes*	Low-salt chicken sausages	0.5 or 1% MP	Meat batter with a higher pH. Lipid oxidation was blocked, and the texture was smoothed out. Enhanced the food’s nutritional value. There is no detriment to hue and sensitivity.	(113)
*F. velutipes*	Goat meat nuggets	2, 4, or 6% MP	Higher mineral and fibre content after cooking. To prevent lipid oxidation, which shortens the shelf life of beef products. The sensory qualities are not harmed.	(44)
*F. velutipes*	Emulsion-type pork sausages	0.5, 1, 1.5, or 2% MP	Elevated pH and WHC at >1% inclusion level. Less fat and moisture seeped out of the sausages. At a level of inclusion of less than 1.5%, there is no noticeable change in colour or sensory qualities. Possible alternative to phosphates for use in meat. Compared to phosphate control samples, they had a softer texture.	(45)
*L. edodes*	Fermented sausages (pork ham 80% and pork fat 20%)	NA	Exhibited greater resistance to lipid oxidation and antioxidant activity. Had more potent antibacterial effects on disease-causing microbes. Flavours and taste were partially enhanced in comparison to the control group after ethanolic extract was added. Scored much higher than the control group on colour, flavour, taste, and acceptability.	(114)
*L. edodes, P. ostreatus, Coprinus comatus*	Sausages	NA	In terms of both the senses and the texture, it was comparable to beef.	(112)
*P. sapidus*	Vegan boiled sausage and meat products	NA	Characteristics of the flavour profile, stability, shelf life, water absorption index, texture, sensory, and colour.	(115)
*Pleurotus eryngii*	Chicken burger	10 or 15% MP	Improvements in the ability to store water. At a 15% inclusion rate, this reduced cooking-related weight loss and storage-related thickness. Increased flavour and softness.	(116)
*Tremella fuciformis*	Pork patties	10, 20, or 30% MP	Superior ability to absorb both liquids and oils. Resulted in a much higher yield when cooked. Has more yellow and lighter tones than the control (64.31–67.23). There was no change in hue, taste, or texture.	(117)

MP, Mushroom powder; NA, Not applicable.

Mushrooms are a potential addition to meat substitutes or used alone as a meat substitute because of their flesh-like properties, umami flavour, and compatibility with other meats. Due to the similarities and harmonious combination of mushrooms and meat are similar and harmonious, consumers prefer meat products that contain mushrooms (35). The incorporation of mushrooms as meat alternatives significantly enhances the functional qualities of mixed meat products. This category includes the ability to retain water, the volume of meat gained after cooking, the texture, the stability of the mixture, the juiciness, the shelf life, and associated qualities. In some cases, there has been a lot of research done on the method of utilising ground mushrooms as a meat substitute (36) (Table 4). These composite foods, in addition to preserving the consistency and flavour of traditional meat-based recipes, are considered both environmentally sustainable and edible. Numerous studies have demonstrated that mushrooms are a suitable replacement or addition for producing beef products that are higher in nutrients (37). Due to their health benefits, beneficial traits, increased nutritional value, enhanced antioxidant activity, and ability to improve the texture and flavour of meat products, mushrooms are attracting the attention of food companies. It has been shown by research that using *Agaricus bisporus* in place of animal fat in beef burgers is a potential technological, nutritional, and sensory strategy (38). Scientific research also exhibited mushrooms as a good substitute for beef. Their high content of dietary fibre, readily digested proteins, and texture, which closely resembles animal meat, explain this. This can lessen the sustainability and health problems associated with eating animal products (39). In addition to providing new options for protein, fat, phosphate, and salt in meat product compositions, mushrooms can improve sensory qualities. Mushrooms can successfully replace animal fat in beef burgers because they maintain moisture and fat, while also reducing the effect of animal fat loss on the burgers’ sensory qualities. In a recent study, the sensory and physical properties of foods such as meatballs, tacos, burgers, and similar dishes that contain meat with mushrooms has been investigated (5). Using mushrooms as meat replacements greatly reduces the consumption of animal meat. This suggest that, mushrooms be included in beef products as workable substitutes or additions that could provide benefits in terms of taste, nutrition, and technology.

**Table 4:** Mushroom-based meat analogues/replacer.

Mushrooms	Ingredients	Product	Impact on product	References
*Agaricus bisporus* (Steamed and roasted)	Egg white replacer (10%, 20 and 30%)	Mushroom or egg White patty	Good hedonic scores except for general acceptance of grilled mushroom patties. Mostly popular are steamed crimini 20% patties.	(118)
*A. bisporus, Pleurotus ostreatus* (7.5 and 10%), dried	Reduction of fat (30 and 50%); Reducing nitrite, phosphates, and salt (50%)	Liver pate (30% fat, 2% salt)	Changes in pH, dietary fibre, protein, and colours; Improvement in adhesiveness and hardness.	(119)
*A. bisporus, P. ostreatus* (2.5 and 5%), dried	Reduction in fat (25 and 50%); Reduction in salt (50%)	Beef-burger (fat 10% and salt 1.2%)	Increased nutritional fibre and protein, lessened hardness increases in comparison to fat-reduced control samples, mild antibacterial impact in pseudomonads, and sensory acceptance, particularly in 2.5% of the mushroom samples.	(76)
*A. bisporus, P. ostreatus* (2.5 and 5%), dried	Reduction of fat (30 and 50%); Reduction in caseinate, phosphates, and salt	Frankfurter (fat 25%, salt 1.5%, sodium 2%, caseinate, phosphate 0.5%)	Changes in texture and colour were noted; *Pleurotus* produced softer samples, *Agaricus* produced darker samples, no antioxidant effect. The mushroom samples were sensory acceptable, scoring higher at the low concentration.	(76)
*A. bisporus* (15 and 30%), cooked	Reduction in fat (25 and 50%); Reduction in salt (37.5 and 75%)	Beef-burger (fat 20% and salt 2%)	Increasing moisture without altering colour or hardness, 15% mushroom showed minimal lipid oxidation, and salt content altered the sensory profile.	(120)
*A. bisporus* (5, 10 and 15%), cooked	Substitution of fat (25, 50, and 75%)	Beef-burger (20% fat)	Overall taste is good, fewer tough and chewy foods, reduced cooking loss, colour change, and 15% strong antioxidant qualities.	(82)
*A. bisporus,* Fresh	Reduction in salt (0–40%)	Chicken-nuggets (1% salt)	Adding eggplant to a low-salt diet (13%) increased cooking yield, shrinking, and firmness. Loss of physical properties when the salt amount was lowered.	(121)
*A. bisporus,* Fresh	Meat substitution (10, 20, 30, 40, 50%); Reduction in salt (25%)	Beef patties	An overall preference, aroma, juiciness, flavour, and salinity were observed in the 20% mushroom formulation.	(109)
*Flammulina velutipes* (winter mushroom) 1% dried	Nitrite/Phosphate (total replacement)	Ground-ham (3 g/kg Phosphates and 48 mg nitrite/kg)	Good-source of nitrite; no total substitute for phosphates in processed meat products.	(122)
*F. velutipes* steam wastes, dried	Meat substitution (2, 4 and 6%)	Goat-meat nuggets	Enhancement in oxidative stability, water holding capacity, absence of detrimental effects on colour or sensual, qualities 4% suggested a replacement.	(44)
*Lentinula edodes* (0–6%), dried	Phosphate replacer	Pork patties (0.5% phosphate)	The shiitake mushroom made things more juicy and less tough and rubbery.	(79)
*L. edodes,* dried	Substitution of meat (25, 50, 75, and 100%)	Pork-meat sausage	Increased moisture, fibre, methionine, glutamic, cysteine, and total phenolic; antioxidant activity decreased during cooking. Light sausage darkening and all formulas were sensory-acceptable, although 25% replacement was optimum.	(123)
*L. edodes* Water extract (5, 12.5, and 20%), water extract	Salt reduction (50 and 75%)	Beef patties (1.3% salt)	The 20% extract was effective as a taste enhancer in a 50% reduction in salt, resulting in improved acceptance in colour, aroma, texture, flavour, and overall impression.	(124)
*P. eryngii* (raw, boiled, deep fried, fried)	Fat Substitution (100%)	Pork sausages (fat 17%)	Loss of 83–90% of fat, gain in protein, moisture, dietary fibre, heat loss, and fluidity flavour, texture, and general acceptability were better when the mushrooms were deep-fried or fried.	(125)
*Pleurotus sapidus* Waste, dried	Meat replacement (10, 20, and 30%)	Chicken patties (65% meat)	A formulation with an ideal concentration of 10% resulted in a significant increase in both hardness and chewiness.	(126)
*A. bisporus, Volvariella volvacea, P. ostreatus* and *Hypsizygus marmoreus,* (5%), dried	Flavour enhancer	Beef paste	Boosts the production of crucial amino acids and stimulates the creation of flavour compounds.	(108)

## 5 Quality aspects of mushroom-based meat products

### 5.1 Effects on physicochemical properties

The quality of meat-based food is influenced by several physicochemical variables, such as chemical composition, pH, emulsion stability, water-holding capacity (WHC), and cooking yield (40). Acidity or alkalinity controls the growth of microbes, which in turn influences the cooking yield, WHC, juiciness, texture, and longevity of many foods (41). Powdered winter mushrooms (*F. velutipes*) increased the pH, WHC, and cooking yield of emulsion-type pork sausages while decreasing the exudation of fat and water (42). The addition of white jelly mushrooms (*T. fuciformis*) significantly increased the cooking yield and oil retention of pork patties and chicken patties with 25% fresh mushrooms increases cooking yield by 81% and moisture retention by 77% (43). Recent studies demonstrate that adding *F. velutipes* stem waste to goat meat nuggets enhances their pH, emulsion stability, cooking yield, and WHC (44). A slight increase in pH was observed when enoki mushroom extracts were added to beef and fish products. This rise in pH after mushroom addition might be explained by the fact that mushrooms have a natural buffering capacity and that these products contain a higher proportion of basic amino acids than acidic ones (45). Researchers found that adding shiitake (*L. edodes*) extracts to fermented sausages caused the pH to decrease during 30 days of storage at 15°C. This may be attributed to the presence of acid-producing lactic acid bacteria in the fermented sausages (46).

Incorporating mushrooms into meat products changes their nutritional profile and chemical composition. Because they contain much higher amounts of protein, minerals, and dietary fibre, mushrooms are responsible for the effects specified earlier (3). A study indicated that beef patties with dry fungus (*P. ostreatus*) added had higher levels of protein, fat, and ash than the raw beef patties. Five chicken patties recipes were tested, each with a different proportion of chicken to grey oyster mushroom stem: control (65%:0%), A (55%:10%), B (45%:20%), C (35%:30%), and D (25%:40%). An analysis was conducted on the chicken patties to determine their nutritional content, physicochemical features, and cooking characteristics. In comparison to the control, the patties made from stems of grey oyster mushrooms had a greater cooking yield and moisture retention rate (43). Additionally, the cooked chicken patties had much less fat after adding dehydrated grey oyster mushrooms (34). Another study found that adding dried *F. velutipes* extracts to goat meat nuggets increased their dietary fibre and ash levels (44). Frankfurters found to had a lower lipid level and a higher dietary fibre content after adding powdered oyster mushrooms (*P. sajor caju*) to chicken meat (8). These studies reveal that various kinds and quantity of mushrooms can affect meat-based food composition during processing. Mushroom powder or extract influences meat’s nutritional, sensory, physical, and chemical attributes.

#### 5.1.1 Effects on the textural properties

Meat-based food’s texture greatly affects its visual appeal and quality characteristics. Semi-solid textures influence the digestion, chewing, and preparation of soft materials with complex structures and compositions, such as meat-based foods. Meat-based proteins have emulsifying and gel-forming capabilities, which affect not just their textural properties but also those of other components, such as minerals and lipids (47). Adding mushrooms to meat-based foods changes their rheological properties. This issue must be considered when producing mushroom-enhanced products. Mushrooms are an excellent substitute for meat due to their firm texture and the flesh-like texture created by their dietary fibre fractions when combined with meat-based foods, resulting in meat-like features. Mushrooms are typically safe to add to meat-based foods up to a certain point without significantly altering their texture (44). The effect of mushrooms on the textural qualities of beef products has been studied extensively. Adding mushroom powder made sausages less chewy, gummier, springier, and firmer (48). Similarly, it was found that adding mushroom extracts made the beef nuggets less solid, resilient, compact, or sticky (44). Studies have demonstrated that the hardness and other texture-related properties of chicken patties decrease when oyster mushrooms account for 25% or 50% of the chicken meat (43). A study found that adding king oyster mushroom to surimi gel-a gel made from cuttlefish meat paste-increased springiness and decreased hardness, cohesion, and gumminess (49). Hence, it exhibits that, in most cases, the addition of mushrooms to meat-based recipes results in tenderised end products. Dietary fibres found in large amounts in mushrooms can create a three-dimensional biopolymer network. The capacity of this network to retain liquids gives beef products a softer texture (50). Significant amounts of mushrooms also lower the concentration of soluble meat-based proteins in beef products, which makes it harder for them to create robust gels.

**Figure 3:**
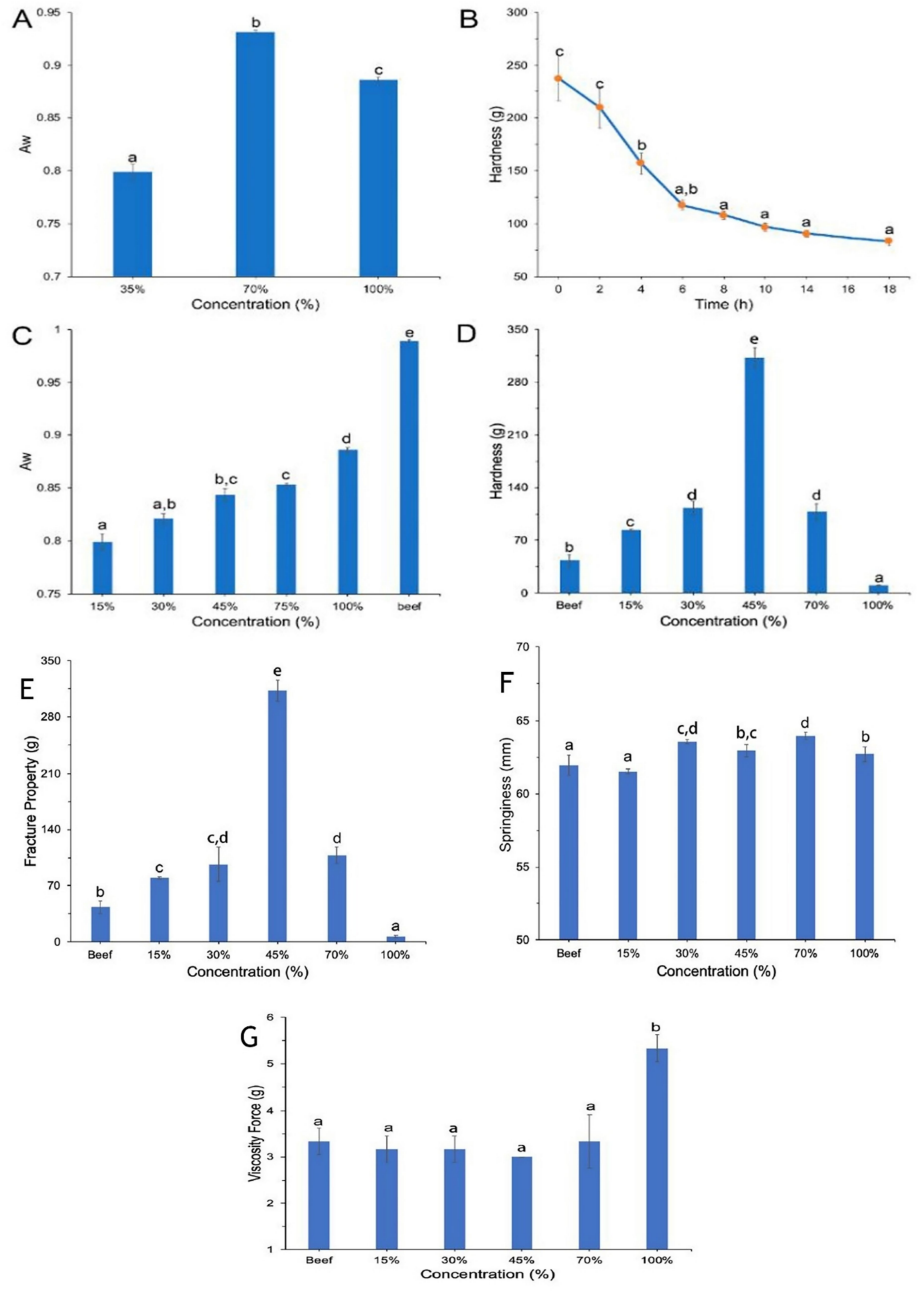
**(A)** Mushroom based sausage with water contents 35–100%, **(B)** hardness of sausage with different rehydration times, **(C,D)** Aw and hardness, **(E)** fracture properties, and **(F,G)** variations in viscosity and springiness of sausage with edible mushroom contents (15–100%) compared with beef. **(A-D)** Reprinted with permission from “The aw of mushroom-based meat analogues with water contents from 35% to 100%; **(B)** The hardness of mushroom-based meat analogues with different rehydration times; **(C)** The aw and **(D)** hardness of mushroom-based meat analogues with contents of edible mushroom from 15% to 100% compared with beef.” by Yuan et al. (1). **(E-G)** Reprinted with permission from “Figure S1: Fracture properties of meat analogues”, “Figure S2: Springiness of meat analogues”, “Figure S3: Viscosity of meat analogues” by Yuan et al. (1), licensed under CCBY 4.0.

A sausage was made using a combination of these mushrooms in equal proportions with *Lentinus edodes, Pleurotus ostreatus*, and *Coprinus comatus* (1). An evaluation of the extrudate’s water activity (a_w_) was conducted after using the ingredients with water contents of 35, 70, and 100%. Because it influences protein mobility, cross-linking, and water absorption, water concentration is critical for extrudate structure. The a_w_ of the sausage was less than 0.85 due to its 35% water content, which is ideal since it prevents the growth of microorganisms and, to a lesser extent, allows for storage and a longer shelf life (Figures 3A–G). As a result, we settled on a 35% water content as optimal, and going forward, we’ll need to hydrate any extrudate that’s less than 45%. Figure 3B shows that by considering the stiffness of the extruded edible mushroom-based meat replacement, the rehydration time was improved in this study. After being submerged in water for 8 h, the extrudate’s hardness stabilised after a gradual drop. It was determined that this duration was ideal for rehydration.

The a_w_ was elevated in proportion to the quantities of mushroom (Figure 3C). With a mushroom content over 45% and a_w_ greater than 0.85, the conditions are favourable for the growth of microorganisms. Following the addition of edible mushrooms, the rehydrated meat substitute formed a water dispersion with solid particles that resembled a soft mud, making it difficult to proceed with any additional processing (1). The texture profile analysis (TPA) was used to assess the variations in structure and textural properties of meat analogues containing varied amounts of edible mushroom and beef. When 15% of mushrooms were added, the meat analogue showed the highest similarity to beef in terms of hardness (Figure 3D). The fracture properties also followed a similar trend (Figure 3E). However, there were no variations in springiness and viscosity across the meat analogues (Figures 3F,G). In terms of its longevity and consistency, incorporating 15% of mushroom into the meat substitute was an appropriate ratio.

**Figure 4:**
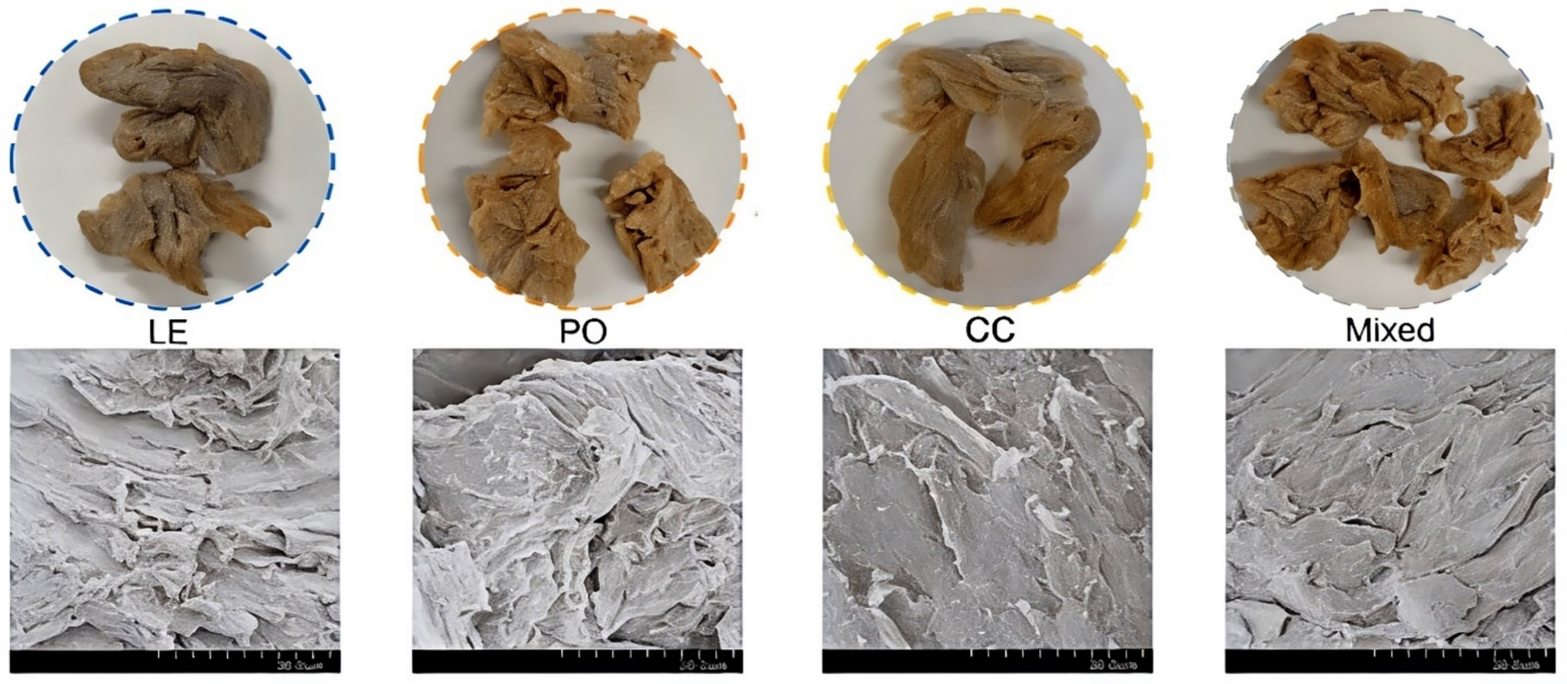
SEM image of meat analogues containing edible mushroom *Lentinus edodes* (LE)*, Pleurotus ostreatus* (PO), and *Coprinus comatus* (CC), and the mixture of all mushrooms (Mixed). Reprinted with permission from “The texture information obtained from photographs of meat analogues containing edible mushroom from LE (blue), PO (orange), CC (yellow) and the mushroom mixture (grey) and corresponding inner microstructure information obtained from SEM.” by Yuan et al. (1), licensed under CCBY 4.0.

The visual characteristics of extrudates made from a variety of edible mushrooms (*L. edodes, P. ostreatus*, and *C. comatus*) was also confirmed (Figure 4). The extrudates from *Coprinus comatus* and *Pleurotus ostreatus* had a clearly defined fibrous morphology and an attractive brightness, but the extrudates from *Lentinus edodes* were lackluster, with several areas of dark spots. SEM images revealed a distinct layered structure in the extrudates. Compared to those made from *P. ostreatus* and the combination, the extrudates that contained *L. edodes* and *C. comatus* were more homogeneous and had a well-structured fibrous morphology. More precisely, the extrudates made from *P. ostreatus* showed a more compact and helical fibre structure, which could explain their higher level of hardness. These findings indicate that both *P. ostreatus* and the combination were not appropriate for protein texturization using this specific processing method (1). Aspects such as storage conditions, appearance, and tactile characteristics influence customers’ acceptance and preference of food products (51). Therefore, the *C. comatus* was highly recommended in the creation of the meat alternative.

#### 5.1.2 Effects on lipid oxidation

Oxidation is an unwanted process since it causes different food preparations to produce unwanted scents and unpleasant odours, as well as colour changes. Lipid oxidation generates a variety of free radicals, including alkyl, alcoxyl, and peroxyl radicals. It is believed that these radicals cause protein oxidation (52). Cooking or heating meat-based foods over 60°C, decomposes the porphyrin ring and releases heme iron. As a result, lipids and proteins may become more oxidised (53). The intricate oxidative processes form a variety of reaction products and result in the loss of vital nutrients. Consequently, the body loses essential fatty acids and amino acids, along with the process generates volatile off-flavours (54). Many factors, including oxygen, heat, light, and transition metal ions, affect the oxidation of proteins and lipids. In muscle-building diets, researchers are looking for and using natural substances that can prevent the oxidation of lipids and proteins. These antioxidants enhance the food’s nutritional profile, shelf life, and overall quality (55).

Numerous naturally occurring antioxidants, such as phenolic compounds, glycosides, polysaccharides, selenium, ergothioneine, tocopherols, ascorbic acid, and carotenoids, are found in mushrooms (56). The primary bioactive substances, which include phenolic compounds (3–11 mg/g) and flavonoids (2.5–4.8 mg/g) are reported from edible mushroom fruit bodies (27). Researchers attribute the antioxidant properties of winter mushrooms (*F. velutipes*) to phenolic substances such as flavonoids, proto-catechuic acid, gallic acid, chlorogenic acid, and quercetin (57). Some research has shown that *B. edulis* methanol extracts exhibit antioxidant properties, such as ascorbic acid (18.7 mg/g d.w.), tocopherols (18.7 mg/g d.w.), and phenolic acids (9.74 mg/kg d.w.). Stems, a byproduct of shiitake (*L. edodes*) mushrooms, contain a broad variety of natural antioxidants (58). Researchers have found strong antioxidant properties in some mushroom species, including *Amanita rubescens, Lepista nuda, Cantharellus cibarius, Hypsizigus marmoreus, Lactarius piperatus, Polyporus squamosus, Mucor circinelloides, Russula cyanoxantha, Agaricus bisporus, Lentinula edodes*, and *Volvariella volvacea*, when extracted from acetone or methanol (59, 60). The high levels of flavonoids and phenolics primarily cause antioxidant activity. The antioxidant characteristics of mushroom extracts (*F. velutipes*) have been shown to slow the oxidation of proteins and lipids in raw beef and bigeye tuna during storage (61). The effectiveness of mixing dehydrated *A. bisporus* powder with salted, cooked ground beef to prevent the oxidation of proteins and lipids during storage have been studied (62). Extensive research has demonstrated that mushroom extract effectively inhibits the oxidation of lipids and proteins. Compared to the control sample, volatile aldehydes produced during storage for 16 days decreased by 99%, and malonaldehyde levels decreased by 88–94%. By the addition of an extract from a button mushroom (*A. bisporus*) to sutchi catfish patties slowed down oxidative changes and made the food last longer (63). Several studies have demonstrated the antioxidant properties of mushrooms and mushroom derivatives. Beef burger patties, dry-fermented beef products, bigeye tuna, kuruma shrimp, and fermented pork sausages may all contain *B. edulis* extract, pulverised white mushroom, or *F. velutipes* extract (8). The inclusion of *Pleurotus ostreatus* in carp burgers increased their antioxidant and taste value (3). It is therefore evident that mushrooms and mushroom extracts are abundant in antioxidants, which by halting the oxidation of lipids and proteins, can extend the freshness and quality of different food products.

### 5.2 Effects on microbial quality

The abundance of macro- and micro-nutrients in meat-based foods promotes the growth of harmful microbes, which causes spoilage. Hence, it is critical to employ efficient methods to prolong the storage duration while guaranteeing the safety of this particular food type (64). There are antimicrobial and antifungal characteristics have been reported in many numbers of mushrooms and their components (65, 66). A variety of substances found in mushrooms, such as peptides, proteins, steroids, anthraquinones, benzoic acid derivatives, and quinolones, are responsible for their antibacterial properties. The fruiting body of the mushroom secretes these substances in order to ensure its own survival (67). As a result, including them in meat-based foods products could potentially enhance their safety and extend their shelf-life. For centuries, people have turned to medicinal mushrooms—specifically, species of *Aleurodiscus, Coprinus, Clitocybe, Daedalea, Marasmius, Merulius, Pleurotus, Polyporus, Poria, Psathyrella*, and *Tricholoma* for their antibiotic needs. This is due to the presence of antibacterial secondary metabolites and immunomodulatory β-glucans in these mushrooms (68)*. Hypsizigus tessulatus, L. edodes*, and *P. ostreatus* extracts had MIC values ranging from 1 to 9 mg/mL against all fungi and bacteria tested. In this study, extracts from two other mushroom species did not perform as well as *L. edodes*. In addition, shiitake (*L. edodes*) mushroom extracts that were separated using organic solvents and supercritical fluids were able to kill pathogenic bacteria like *Staphylococcus aureus* and *S. pyogenes*. Isolates that were separated using only supercritical fluids were able to kill *Bacillus cereus* and *Micrococcus luteus* (69). Researchers have shown that extracts from the *Pleurotus florida* mushroom, as an alternative to traditional antibiotics, strongly suppress the growth of both gram-positive and gram-negative bacteria (70). As a natural preservative, mushrooms or extracts from them can prolong the shelf life of foods through the inhibition of the growth of bacteria that cause them to decay. Sutchi catfish patties made with button mushrooms reportedly have a much longer shelf life due to the mushrooms’ antimicrobial properties (71). Another study demonstrated the particularly potent antibacterial capabilities of shitake (*L. edodes*) extracts in fermented sausages, where they inhibited the growth of bacteria such as *S. aureus, Listeria monocytogenes,* and *E. coli* O157, thereby extending the sausages’ shelf life (72). A methanolic extract from *Boletus aereus* can efficiently suppress poisoning bacteria like *S. aureus, L. monocytogenes, E. coli*, and *Salmonella Typhimurium* in pig flesh (8). The results of these investigations suggest that enhancing the flavour and texture of meat-based foods (such as fish or meat) with mushrooms can increase their safety and storage life.

### 5.3 Effects on sensory attributes

The sensory qualities of food products primarily determine their quality and attraction. Visual presentation, taste, consistency, and oral processing influence the overall sensory perception of meat-based foods (73). The physicochemical properties of meat and fish products are altered when edible mushrooms are added, and these alterations are quantity and type dependent. Because of this, their sensory qualities are changed (15, 74). The pork sausages formulated with 1% winter mushroom powder in an emulsion-type got higher sensory scores in terms of texture, flavour, and acceptability compared to sausages containing 2% (45). Chicken patties incorporating oyster mushrooms at a proportion of 25–50% exhibited comparable flavour and sensory evaluations to the all-meat patties (75). Preliminary studies indicated that adding 25% oyster mushrooms to meat patties did not change their flavour or texture (76). Taste intensity of 25% reduced-salt ground beef tacos was unaffected by the inclusion of 80% white button mushrooms (77). This study indicates that it is feasible to preserve the ideal flavour characteristics of beef products even when using substantial amounts of mushrooms. The high quantity of free amino acids in mushrooms is likely the cause of this impact; these acids produce the desired umami, sweet, and bitter flavours seen in meat (78). Edible mushrooms have a delicious umami taste that enhances their flavour and makes them versatile for several culinary purposes. Adding Shiitake mushroom powder to pig patties at different concentrations (2, 4, and 6%) improved their texture, moisture content, taste, and overall acceptability (79, 80). When different amounts (30, 40, and 50%) of king oyster mushroom were added to cuttlefish (*S. esculenta*) paste, the overall acceptance scores were much higher than with the control paste (81). By adding mushrooms or mushroom extracts like enoki mushroom powder (2, 4, and 6%) to mutton nuggets and ground white jelly mushrooms (10, 20, and 30% pulverised) to pig patties, the taste and texture of these meat products stay the same or get better (44).

**Figure 5:**
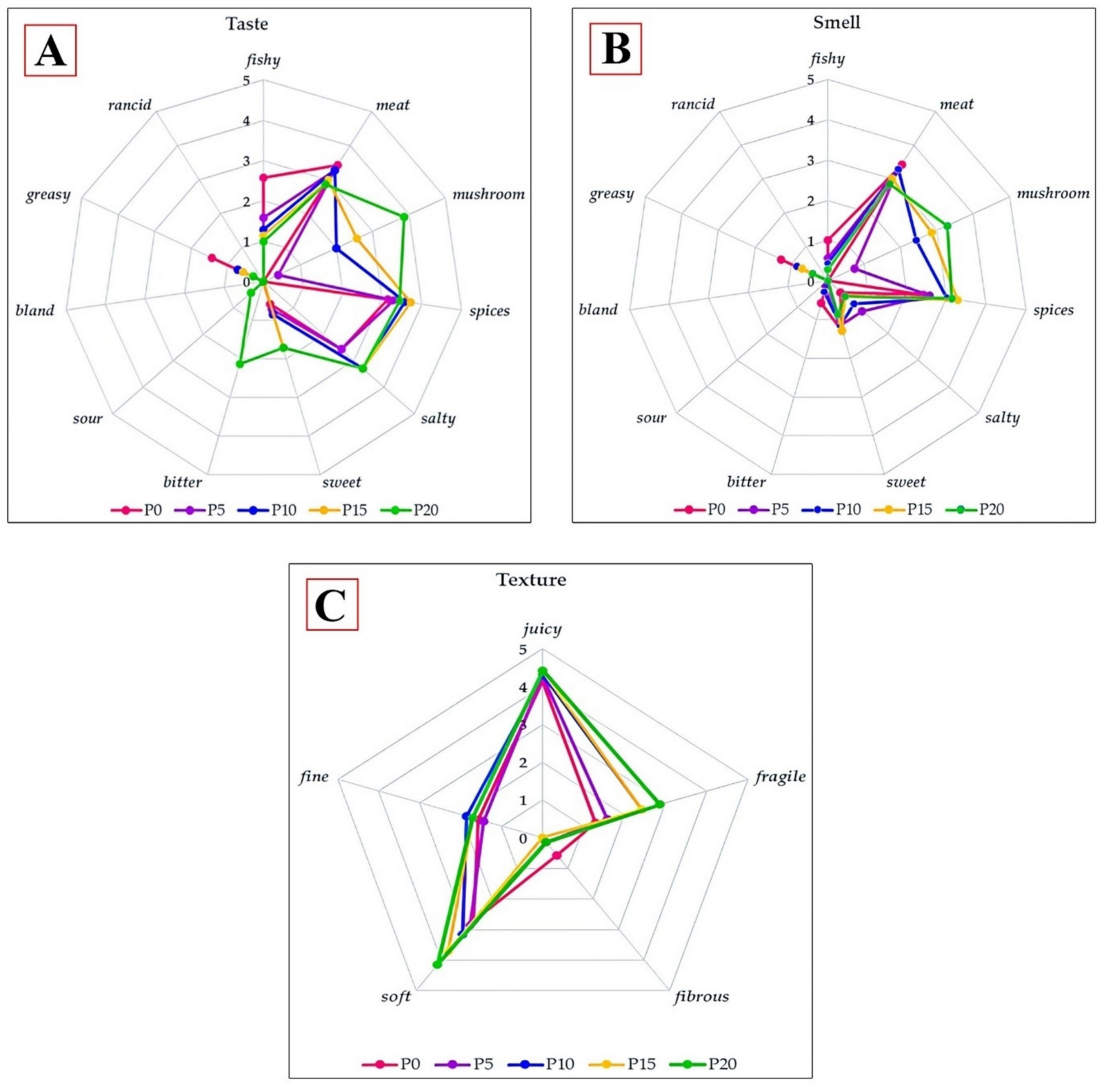
Sensory profile (**A**—Taste, **B**—Smell and **C**—Texture) of fried burgers with/without oyster mushrooms. Concentrations of mushroom powder-0% (P0), 5% (P5), 10% (P10), 15% (P15), and 20% (P20), and 0-imperceptible, 1-very weak, 2-weak, 3-moderate, 4-clear, and 5-very clear are sensory qualities were assessed on a range of scales. Reprinted with permission from “The taste profile of fried burgers with and without oyster mushrooms on a 6-point hedonic scale (0—imperceptible, 1—very weak, 2—weak, 3—moderate, 4—clear, and 5—very clear).”, “The smell profile of fried burgers with oyster mushrooms and without them on a 6-point hedonic scale (0—imperceptible, 1—very weak, 2—weak, 3—moderate, 4—clear, and 5—very clear).”, “The texture profile of fried burgers with and without oyster mushrooms on a 6-point hedonic scale (0—imperceptible, 1—very weak, 2—weak, 3—moderate, 4—clear, and 5—very clear).” by Tokarczyk et al. (3), licensed under CCBY 4.0.

A recent study conducted a sensory assessment of carp burgers, comparing samples with/without the addition of oyster mushrooms. In addition to the carp burgers, several concentrations of mushroom powder-like 0% (P0); 5% (P5); 10% (P10); 15% (P15); 20% (P20), were added, and the sensory qualities were assessed on a range of scales, including (0-imperceptible; 1-very weak; 2-weak; 3-moderate; 4-clear; 5-very clear) (3). Through a thorough evaluation of the flavour of fried burgers, it was demonstrated that the perception of their distinct qualities varied depending on the inclusion of oyster mushrooms (Figure 5A). In the control sample (P0), where the mushroom was not involved, the taste of the meat was noticeably (*p* < 0.05) predominant, followed by the flavours of spices and greasiness, while the spice flavour was relatively prominent. When oyster mushroom was added to the samples, the flavour of the mushroom became considerably more noticeable (*p* < 0.05) as its concentration grew, going from extremely weak (P5) to clear (P20). The flavour of the meat in these samples, akin to salty, was moderate and slightly above average (3). The study has demonstrated that including mushrooms at concentrations of 10 and 15% enhanced the flavour of burgers and accentuated their saltiness (82). In sample P0, the intensity of spices was significantly greater compared to all other samples (*p* < 0.05), but the intensity of fish was significantly lower (*p* < 0.05). The burgers, containing a 20% proportion of oyster mushrooms, exhibited a noticeable (*p* < 0.05) augmentation in taste characterised by a subtle bitterness. All assessed samples were devoid of any rancid flavour. Based on the evaluators’ assessment, including mushrooms up to a 15% proportion had a statistically significant (*p* < 0.05) and favourable impact on enhancing the taste characteristics of carp burgers.

The aromatic analysis of fried burgers incorporating oyster mushrooms demonstrated that three distinct attributes, specifically mushroom, meat, and spices, exerted a substantial influence on their composition (Figure 5B). Once introduced, the mushroom aroma became more noticeable in the samples, ranging from a very faint intensity (P5) to slightly higher than moderate intensity (P20). There is a considerable (*p* < 0.05) impact on its detectability when 10% of mushrooms are added. Both the meat and spice odours were discernible, with scores ranging from 3 to somewhat higher, on the discriminant scale. The assessors detected a pronounced meat aroma and a faint spice scent in sample P0. However, the capacity to identify this odour did not exhibit a statistically significant disparity in comparison to its intensity in other samples (3). Nevertheless, the fish odour in this specific sample was fairly weak, and the mushroom’s influence was almost insignificant. The testers found no sour or rotten smells in the samples, suggesting the technology used fresh ingredients.

Upon analysing the texture of the finished carp burgers, it was observed that regardless of whether oyster mushrooms were included or not, the level of juiciness perceived in all samples was significant, comparable, and insignificant. As the percentage of mushrooms increased, the samples exhibited a greater degree of sensitivity and softness (3). The juiciness and softness of the samples with 15 and 20% of mushrooms show a significant increase (Figure 5C). All of the samples, except for the control sample, which did not include oyster mushrooms, were not fibrous. The addition of mushrooms to carp burgers in quantities of 10 and 15% considerably enhanced their texture. Concerning the sample containing the highest proportion of mushrooms 20%; the texture was noticeably more tender and deviated significantly from the desired standard, being excessively delicate and moist. The control sample, devoid of mushrooms, exhibited reduced tenderness and delicacy, leading to somewhat diminished acceptability in comparison to the tests that incorporated mushrooms (P10 and P15). The texture results obtained have been validated by the research conducted by many scientific study (82). Therefore, the addition of mushrooms to the burger improves its texture by introducing a subtle tenderness and decreasing its firmness. Furthermore, mushrooms possess a remarkable ability to retain moisture within the product, resulting in a more succulent texture and minimising any loss of volume, therefore preserving their desired form.

#### 5.3.1 Effects on appearance

Meat-based food opacity, colour, and surface sheen affect consumer purchasing preferences because of their quality and freshness. Mushrooms alter the appearance of this food in a variety of ways. Because mushrooms and meat or fish are different colours, mushroom blends will look different (63). Mushroom extracts may also have particles of different sizes and shapes than meat-based foods, causing them to change their appearance. Mushrooms also include antioxidants and other substances that may prevent meat-based foods colour changes (83). The winter mushroom (*F. velutipes*) extract can change the colour and met-myoglobin concentration of minced beef and bigeye tuna during cold storage (45). The amount of haemoglobin in the beef products was considerably reduced by the mushroom extracts. This meant that the beef and tuna items kept their colour for up to 12 and 7 days of storage, respectively, but the control samples (non-mushroom) barely made it 6 and 2 days. Ergothioneine, responsible for the colour-stabilising activity of the mushroom extract, slowed down the rate of met-myoglobin production, known to cause discoloration in meat-based foods. Moreover, studies have demonstrated that an ergothioneine extract from edible mushrooms can maintain the red colour of tuna and yellowtail fish meat during cold-storage (49). Another study found that while cooked chicken patties with 50% oyster mushroom added did not change in redness (a^*^), they did decrease in lightness (L^*^) and yellowness (b^*^). Similarly, adding up to 20% *A. bisporus* mushroom did not significantly alter the appearance of beef patties (82). While shiitake mushroom powder added to frankfurters did not alter their initial colour, it did make them more yellow while preserved. According to a study, the lightness (L^*^) of a cooked beef taco decreases with increasing white button mushroom concentration (25–75%). Furthermore, the addition of 75% mushroom resulted in a lower redness (a^*^) in the taco meat compared to the all-meat control samples. These effects may have originated because the mushrooms reduced the myoglobin content of the finished products and were darker than the meat products. White jelly mushrooms (*Tremella fuciformis*) were found to somewhat decrease the redness and increase the yellowness of cooked pig patties, which contradicts earlier study. This could be due to the mushrooms’ clear white colour (84). Mushroom effects on meat-based foods are typically determined by their initial colour and any potential physical or chemical interactions between these two.

## 6 Consumer acceptance to mushroom-based meat analogues

It is essential to have a comprehensive understanding of the client’s perspective in order to produce meat analogues. Discovering the elements that excite and demotivate consumers is the first step in the process of building future meat analogues. Several factors significantly influence consumers’ purchase and consumption of meat analogues. These qualities comprise taste, satisfaction, price, brand, health and wellbeing, environmental effect, animal welfare, and so on (29, 85). According to the findings of the research, the elements that influence customer choice are, in descending order of importance, price, environmental impact, flavour, health, organic, vegetarian, and other aspects (86). Different aspects of the consumer, including as their gender, age, geography, and level of education, play a vital role in determining whether or not they will purchase meat analogues. An international study that was carried out in the United States of America, China, and India discovered that the purchase of meat substitutes was influenced by a variety of factors in each of the three countries (32, 74). Indicators that were trustworthy in the United States were attraction, limited dislike, and passion for the topic. Among the most important characteristics that were used to predict outcomes in China were general health, sustainability, beauty, and taste. In contrast, the factors of wholesomeness, sustainability, necessity, and excitement were found to play a major influence in predicting the purchasing behaviour of meat analogues in India (87) According to the findings of a number of research, there is a significant connection between the dietary habits of consumers and their propensity to consume alternatives to meat. “Carnivores (traditional eaters), semi-vegetarians (flexitarians), and vegetarians” were the three fundamental groups that were identified under that framework (88). Edible mushroom-based meat substitutes have the potential to appeal to traditional diners, flexitarians, vegans, and vegetarians due to their umami flavour, appealing texture, and capacity to fulfil the requirements for protein.

There are serious issues with eating mycoprotein, especially when it comes to the possibility of allergic reactions and the generation of microbial toxins (1). However, compared to typical allergenic foods like soy, peanuts, and eggs, Finnigan et al. (89) found that gastrointestinal responses associated with mycoprotein consumption are much lower. Traces of fumonisins (8.60 μg/kg) can be produced by *F. venenatum*, and concentrations rise in the production medium when there is significant moisture content (89). Similarly, *Aspergillus* and *Penicillium* can contaminate some foods with citrinin, a carcinogenic mycotoxin. Consumer preferences are significantly influenced by sensory qualities, sustainability, healthiness, and naturalness. Purchase decisions are further influenced by age, meal environment, and dietary habits. On the other hand, consumer acceptability may be hampered by food technology neophobia, perceived advantages, and mould associations (90).

## 7 Sustainability aspects

Mushroom-based meat substitutes are becoming quite popular as healthy, ethical, and environmentally friendly alternatives to meat. Their rise is changing the way people choose food and how they think about it, especially those who care about the environment and their health (26). They bring people together by offering high-protein meals that vegetarians, vegans, and flexitarians can eat without killing animals. These also help make food fairer by making it easier and cheaper to grow mushrooms in the area. This gives small-scale farmers and business owners new chances, especially in rural areas. Mushroom meat is becoming more popular because it combines eating meat with being a vegetarian (27). This promotes sustainable food practices and makes it more appealing to a wider audience. Also, they make it less necessary to rely on large-scale livestock production, which raises a lot of ethical and environmental issues, such as animal welfare and greenhouse gas emissions. Their low environmental effect encourages a more responsible way of thinking about consumers and helps efforts to make the world more sustainable (15). Culinary innovation makes things more socially acceptable by making them taste and feel like animal meat, which makes customers happier. Awareness and instructional programmes reduce suspicion and spread the word about their benefits. Mushroom meat products are a type of cuisine that people are starting to eat more. They are part of a new social movement that promotes rational eating, ecological balance, and an ethical food culture (17, 35).

Mushroom meat addresses the major concerns with animal welfare, environmental stewardship, and responsible consumerism in an ethical manner. Mushroom meat bypasses the ethical dilemma of killing animals and significantly lowers the environmental footprint associated with meat production (26, 91). Nevertheless, ethical challenges remain, such as transparency in manufacturing methods, the use of synthetic ingredients, and equitable distribution. Corporate monopolisation of plant-based technology could exclude small manufacturers and drive-up prices, thus limiting access. Ensuring ethical sourcing, fair trade, and inclusive economic structures is crucial. As the mushroom-based meat industry grows, it will be vital to address food safety, security, and ethical issues to ensure long-term public acceptance and sustainability (92).

## 8 Global market and adaptation

At present, meat analogues are primarily produced using vegetable proteins, such as soybeans, peas, and wheat. Their popularity can be attributed to their easy accessibility, affordability, widespread usage, and nutritional value that is comparable to animal proteins. The efficient use of plants as primary resources for the production of meat alternatives for consumption has led to significant advancements in their market accessibility. For example, beyond meat and impossible foods are leading American companies that are at the forefront of fake meat development. Both companies have secured patents in many countries and formed collaborations with various prestigious domestic and international restaurant brands and e-commerce platforms, including KFC, Starbucks, Subway, Be & Cheery, Jindinxuan, Heytea, Lawson, Tmall, and Jingdong. The number of local Chinese companies producing plant-based meat substitutes is rapidly increasing, providing a broader selection of products, such as Plant Plus’s vegetarian meat dumplings, Be & Cheery’s vegetarian meat Zongzi, and Except Meat’s vegetarian beef meatballs. Nevertheless, the availability of mushroom-based meat substitutes is limited (93, 94). The market for edible mushrooms is booming, and there is an increasing desire for plant-based alternatives to meat. Consequently, there is a growing fascination with creating meat substitutes using edible mushrooms as the main component. Although there have been many investigations into the use of edible mushroom protein as a substitute for meat, the advancement in bringing these goods to market is still in the early stages. Compared to other vegetable protein meat substitutes, the market now offers a limited selection of edible mushroom protein meat substitutes. Nevertheless, the market continues to provide plant-based meat substitutes derived from mushrooms. Food items made from certain types of mushrooms include pickled fish fillets made from *Hericium erinaceus* (found in plant diaries) and mushroom meat snacks made from *L. edodes* (found in Vesta). Xuerong Bio, a publicly traded Chinese edible mushroom company, intentionally expanded into the edible mushroom deep processing sector in 2020. Other prominent competitors in the sector quickly followed them after their collaboration with other food companies to develop edible mushroom protein revolutionised meat substitutes (85). The increasing awareness among consumers of environmental difficulties and health risks linked to meat consumption is the main driver behind the market demand for meat alternatives. Various global enterprises (including Shroomeats, USA; Green Monday, Hongkong; Myco Technology, USA; Innomy, Argentina; Meati foods, USA; Mushlabs, Germany, Europe; Chinova Bioworks, Canada and many more) are rapidly expanding their presence in the market for meat substitutes made from mushrooms, are listed data in Table 5. Due to mushroom mycelium’s quick growth, resilience, and ability to convert growth material into nutrient-rich byproducts, several start-ups employ solid-state fermentation and submerged cultivation methods. Because of its elevated protein concentration, it can function as an alternative protein source for both human consumption and animal feed (95). Besides proteins, biomass contains vitamins, minerals, amino acids, and carbohydrates. Studies have shown that the utilisation of mycoproteins obtained from mushrooms does not cause any immediate or long-term health problems (96). The nutritional value of cow, pea, mushroom, and soy burgers was assessed (97). Mushroom-and-soy burgers had the highest nutritional standards. Mushroom mycelium is a unique type of protein, similar to that found in plants and animals (98). The extraction of mushroom mycelia from food and agro-industrial waste represents a novel approach to producing a protein substitute that is more nutritious than animal protein. In recent years, the resuscitation of enterprises that utilise mycelium and fungal proteins has been due to their advantageous nutritional, textural, sensory, and sustainable features. Several companies are utilising mushrooms as a primary component to manufacture meat substitutes. Given the exceptional nutritional properties and the business incentives, it is logical to expect future growth in the market for meat alternatives derived from mushrooms or mycelia.

**Table 5:** Mushroom based meat products in global scenario.

Company	Product	Mushroom and other ingredients	Special features
Shroomeats USA https://www.shroomeats.co	Meatballs, ground meat, burger, patties	Sunflower oil, shiitake mushroom, pea protein, salt, pepper, potato flour	Vegan, gluten free, soy-free, all natural.
Green Monday Hongkong https://greenmonday.org	Omni-pork	Sunflower oil, canola oil, fermented pea, rice protein, potato starch, sugar, and soy	This product is devoid of cholesterol, hormones, and antibiotics. It contains 86% less saturated fat and 66% fewer calories compared to actual pork.
MyForest™ Foods Co. USA https://myforestfoods.com	Whole cut (plant based) meat (like bacon and steaks)	Growing mushroom mycelium makes blocks that are made of meaty fibre and protein-rich food.	High nutritional value, does not contain GMOs or allergens, is eco-friendly, and has all essential amino acids.
Myco Technology USA https://www.mycoiq.com	FermentiQ™	Shitake (mycelium) blend with pea-rice through fermentation	Full protein, better taste properties, easier digestion, better functional properties, no allergens, and lower antinutritional properties.
Moku USA https://mokufoods.com	Plant based jerky	Sunflower oil high in oleic acid, mushrooms, and coconut aminos	Jerky that tastes like meat made from clean, allergen-free plant-based ingredients.
Moving Mountains UK https://movingmountainsfoods.com/	Plant based meatballs	Oat fibre, sea salt, starch, ethyl cellulose, oyster mushrooms, rice, vegetable oil, and vegetable protein	Plant protein, 100% free of hormones, antibiotics, and GMO products, and natural ingredients.
Hooray foods USA https://www.hoorayfoods.com	Plant based bacon	The ingredients are liquid smoke, beet juice concentrate, maple syrup, salt, calcium carbonate, shiitake mushrooms, and coconut oil.	Very little processing, gluten-free, made from plants only, dairy-free, and soy-free.
Fable food Co. Australia https://fablefood.co/	Fable patties, burger	Liquid smoke flavour, shiitake mushrooms, yeast extract, onion powder, tomato powder, rice, salt, gluten-free soy sauce, tapioca starch, garlic powder, and coconut oil.	The thick texture, fleshy fibres, and umami flavours of shiitake mushrooms make them taste and feel like meat. Good for your immune system and full of antioxidants.
Pan’s (Panco Foods) USA https://www.mushroomjerky.com	Mushroom jerkyPan’s	Salmon, coconut sugar, Himalayan pink salt, avocado oil, chia seeds, and organic dried shiitake mushrooms	It’s vegan, gluten-free, vegetarian, Kosher, soy-free, paleo-friendly, high in fibre and vitamin D, plant-based, and made with organic ingredients.
Botanic bites Netherlands https://www.botanicbites.com/	Bourguignon, burger, rescue balls	Sous vide-cooked oyster mushrooms marinated in a variety of spices, tomatoes, coconut milk, and red wine	Avoiding the deterioration of food’s natural nutrients and flavours is the goal of sous vide cooking.

Mushroom-based meat alternatives have attracted attention as environmentally and friendly alternatives to traditional animal meat. However, their growing popularity poses serious problems with regards to food safety, food security, and moral issues (13, 99). From a food safety aspect, it is crucial to ensure the sanitary cultivation, processing, and storage of mushrooms. Mushrooms are highly perishable and susceptible to microbial contamination such as moulds, bacteria, and mycotoxins if not handled properly (100). Quality control during processing is necessary to prevent foodborne illnesses. Some mushroom types also carry allergenic risk, which requires specific labelling to warn consumers, especially sensitives. Mushrooms grown for meat analogue uses are to be closely tracked to avoid mix-ups with unsafe wild types that can pose serious health risks (15). Mushroom-based meats offer substantial advantages in food security. Mushrooms may be produced on farm by-products and in indoor spaces with low resource requirements, making them an attractive choice for protein-consumption diets in food-insecure regions (101). Their short production cycles and tolerance to most environments make year-round supply and localised production possible, potentially reducing dependence on traditional animal industries. This can benefit smallholder farmers, reduce costs, and increase access to high-quality food, thus helping to achieve world food resiliency (101, 102).

## 9 Future outlook

Currently, there is a significant trend of individuals embracing mushroom-based meat products. However, there are many problems that arise during the entire process of cultivating and commercialising mushrooms. Mushroom producers encounter several problems, such as insufficient availability of spawn at the required time, unfavourable weather conditions, absence of cold storage facilities, limited marketing opportunities, and the misconception that mushrooms are non-vegetarian (15, 101). The primary obstacle lies in ensuring a consistent and abundant supply of edible mushrooms throughout the entire year. The successful marketing of mushroom-based meat products requires a substantial supply of edible mushrooms. When formulating meat-based or meat substitutes employing mushroom mycelia, it is extremely important to ensure purity. During the large-scale cultivation of mushrooms for commercial purposes, it is typical for other fungus to infect or grow alongside them. Ensuring or acquiring the desired grade of mushroom blend products is a really demanding task. The lack of mushroom-based meat food processing enterprises equipped with advanced technologies may pose significant obstacles in industry (35).

Food products made from mushrooms are becoming more and more popular. People are increasingly choosing meat substitutes made from mushrooms over meat. To satisfy the demand, a wider range of meat products made from mushrooms is therefore required. The ideal quantity of raw edible mushrooms needed has a direct impact on edible mushroom development and production (26, 35). Both the agricultural sector and the state of the economy could undergo a major change as a result. Commercially speaking, there is a growing demand for meat processing businesses based in mushrooms in order to satisfy the expanding demand for a variety of meat products. It will ultimately function as a platform for the growth and development of new food processing businesses. Additionally, the prevalence of many lifestyle disorders will decline if actual meat (animal-based) products are replaced with meat derived from mushrooms (26). Knowing consumer expertise is important in this context because it is an additional factor in deciding whether or not to include mushrooms in meat products (13). Based on this premise, academic and industrial efforts should focus on making mushrooms more popular as novel ingredients in the creation of meat products with a more environmentally friendly and healthful profile.

The world’s population is expected to increase by one-third from 2015 to 9.7 billion people by 2050, posing serious problems to food production (103). Researchers throughout the world are always looking for sustainable, high-protein, and health-promoting meat substitutes. Because of their natural umami flavour, meaty texture, and high protein content, essential amino acids, polysaccharides (β-glucan and chitin), vitamins, minerals, polyphenols, antioxidants, and medicinal qualities, edible mushrooms may be a viable healthier meat substitute (104). The only vegetative source of vitamin D, mushrooms are also known for their therapeutic potential. They are thought to have a number of health-promoting qualities, including antitumor, antioxidant, immunomodulatory, anti-microbial, anti-inflammatory, cardiovascular-protective, anti-obesity, anti-diabetic, anti-fungal, and anti-cancer effects (105). In addition, mushroom farming is more ecologically sustainable than meat production, leading to reduced environmental impact. Mushrooms are increasingly being used in food innovation, especially in the creation of meat analogues, as customer demand for ethical, sustainable, and healthful food alternatives rises (15). They are a key component of future food solutions due to their numerous culinary and food technology applications, which not only improve health but also help preserve the environment. The global trend towards more sustainable and health-conscious diets is driving a shift in consumer preferences and industrial practices, which is reflected in the move to products based on mushrooms (106). Mushrooms are set to play a major part in the future of food due to ongoing improvements in food technology and rising consumer acceptability.

## 10 Conclusion

The demand for plant-based meat alternatives is expanding, providing consumers with an alternative to meat while also increasing public awareness of the harm that consuming meat causes to the environment and human health. When looking for an alternative source of animal-derived meat products, it is important to carefully analyse key elements including texture, taste, colour, flavour, and entire sensory acceptance to make sure they fulfil the demands of consumers. Mushrooms, with their umami flavour and meat-like texture, can be used as a meat substitute or as an extended to create healthier meat substitutes. Furthermore, mushrooms have higher-quality protein than other plant-based proteins, are easy to grow, abundant in nutrients (vitamins, protein, minerals, polyphenols, etc.), and low in fats and calories. Mushrooms’ higher fibre content also helps to improve the texture of meat substitutes. Due to their numerous health benefits and meat-like texture, mushrooms can serve as eco-friendly meat alternatives in the production of functional foods like sausages, nuggets, and patties. Nevertheless, there remain abundant opportunities and obstacles for further exploration in the realm of improving sensory attributes, refining processing methods, evaluating customer contentment, and harnessing the potential of various medicinal mushrooms as substitutes for meat. As a result, they have a vital role in developing meat-based food products that have improved nutritional and sustainable properties. This review includes important insights into the creation of meat-like, sustainable functional foods made from mushrooms, and it serves as a thorough resource for furthering research in the new area of alternative sources of protein.
